# A Blessing or a Curse? Exploring the Impact of Environmental Regulation on China’s Regional Green Development from the Perspective of Governance Transformation

**DOI:** 10.3390/ijerph19031312

**Published:** 2022-01-25

**Authors:** Xianpu Xu, Xiawan Li, Lin Zheng

**Affiliations:** School of Business, Xiangtan University, Xiangtan 411105, China; lxwxtu20@163.com (X.L.); zlxtu517@126.com (L.Z.)

**Keywords:** green total factor productivity, environmental regulation, governance transformation, the U-shaped relationship, global Malmquist-Luenberger index

## Abstract

China’s rapid economic growth has caused serious problems, such as environmental pollution and resource exhaustion. Only by improving the green total factor productivity (GTFP) can China’s economic development get out of the dual dilemmas of environmental degradation and resources exhaustion. Although environmental regulation helps to improve China’s productivity, its impact on GTFP is still controversial and deserves careful investigation. In this context, this study adopts the global Malmquist-Luenberger productivity index to measure the GTFP change of China’s 30 provinces over the period of 2003 to 2017 and then it uses the fixed-effect dynamic panel model to investigate the impact of environmental regulation on GTFP from the perspective of governance transformation. The results show that: (1) there is a nonlinear U-shaped relationship between environmental regulation and GTFP, indicating that the Porter hypothesis is verified in China. More notably, the values of environmental regulation are still located on the left side of the U-shaped curve at present, which means that the promotional effect of environmental regulation on GTFP has not been realized fully. (2) The U-shaped relationship shows significant regional heterogeneity. The western region demonstrates the highest level of significance, followed by the eastern region. However, the U-shaped relationship is insignificant in the central region. (3) Governance transformation can not only significantly improve GTFP but it can also accelerate the realization of the Porter hypothesis by inspiring the innovative enthusiasm of enterprises, which means that governance transformation can contribute to the achievement of the improved effects of environmental regulation on GTFP. (4) R&D investment can significantly improve GTFP, where the impacts of trade openness and factor endowment were significantly negative and the influence of foreign direct investment was not significant. These conclusions provide a good reference point for optimizing the relationship between the government and the market, as well as promoting regional green and high-quality development in China.

## 1. Introduction

**Figure 1:**
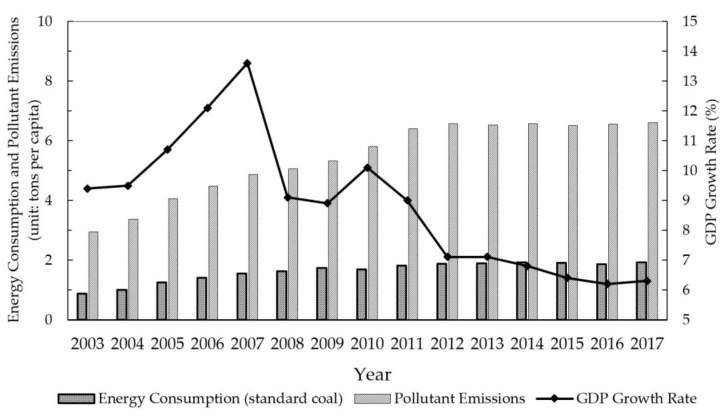
Trends of China’s energy consumption, pollutant emissions, and GDP growth. Source: author’s calculation with data from China Statistical Yearbook (2004–2018).

Since the reform and expansion in 1978, China has achieved remarkable economic growth achievements which benefited greatly from the large scale of the government’s leading investment and factor input [1,2,3]. However, extensive economic development, at the cost of high energy consumption and high emissions, has caused severe problems of resource exhaustion and environmental pollution, which have seriously affected the sustainable growth of China’s economy, as shown in Figure 1. Currently, as the world’s second-largest economy, China is facing the dual pressure of economic development and environmental protection. Meanwhile, China’s economy has entered a “new normal” period, shifting from high-speed growth to high-quality development and total factor productivity (hereafter, TFP) has become the core indicator to measure the degree of high-quality economic development [4,5]. Because the traditional measurement of TFP ignores energy input and does not consider the negative externalities caused by environmental pollution, it is easy to bring about a measurement bias and it cannot truly reflect the quality of economic development [6]. Green total factor productivity (hereafter, GTFP), namely, the total factor productivity when considering environmental protection and economic efficiency, has attracted widespread concerns at home and abroad [7,8]. Therefore, the improvement of green total factor productivity and the promotion of a green economic transformation have become research hotspots in recent years [9,10,11].

With the increasingly sharp contradiction between the environmental carrying capacity and green economic development, strengthening environmental regulation has gradually become a global trend, and China is no exception [12,13,14,15,16]. Faced with severe environmental pressures, Chinese policy makers have formulated a series of strict environmental regulatory policies to address pollution problems. Especially after the 18th CCP National Congress, the central government of China began tightening environmental pollution control, with the enforcement of the new Environmental Protection Law in 2015 and the Thirteenth Five-Year Plan for Ecological Environment Protection in 2016. In addition, 31 provinces in mainland China had been all covered by the central environmental protection supervision system by 2017 and the goals of achieving Ecological Civilization and Beautiful China were also clearly put into the Constitution in 2018. Currently, one of the main objectives of environmental regulation in China is to establish a green and low-carbon circular economic system, in which the driving force of economic growth is changed from investment to innovation [17,18]. In this context, the goal is to reverse the negative environmental impact of economic development and explore the realistic path of green transformation. In view of this, by investigating the effect of environmental regulation on GTFP and its mechanisms, this paper is expected to provide references for formulating reasonable environmental regulation policies for improving GTFP, as well as having important practical significance on the promotion of the high-quality development of China’s economy.

It is undeniable that the rapid development of China’s economy stems largely from the reform and innovation of the economic system [7,19]. In the process of more than 40 years of reform and expansion, the Chinese government has always adhered to the guidelines for establishing a modern market economic system. The role of the market mechanisms in resource allocation has gradually increased, realizing its fundamental transformation from the auxiliary position, to the basic position, and then to the decisive role [20]. The 19th CCP National Congress in 2017 further proposed to speed up the establishment and improvement of the socialist market economic system. Therefore, the relationship between the government and the market, namely, the governance changing from administrative governance to economic governance, is constantly adjusted [21]. Obviously, the role of governance transformation in promoting sustainable economic growth cannot also be ignored. However, few scholars have deeply explored the growth effect of governance transformation from the perspective of GTFP [22]. As a big developing country, China continues reforming towards the socialist market economy. The relationship between governance transformation and GTFP, as well as whether governance transformation can accelerate the dividend effect of environmental regulation on GTFP, are theoretical and practical issues worthy of in-depth exploration [23,24,25].

This paper mainly aims to explore the effect of environmental regulation and governance transformation on GTFP in China. Specifically, based on the theoretical analysis, we use Chinese provincial panel data from 2003 to 2017 to empirically investigate the impact of environmental regulation and governance transformation on GTFP, which is analyzed by constructing a dynamic panel data regression model and using a system generalized moment estimation method. The results showed that environmental regulation inhibited the increase in GTFP in the short term but promoted the growth of GTFP in the long term, and there was a nonlinear U-shaped relationship between environmental regulation and GTFP in China. Meanwhile, the impact of governance transformation on GTFP was significantly positive and, therefore, governance transformation can accelerate the realization of the Porter hypothesis, which reflects the promotional effect of environmental regulation on GTFP. In addition, the results also showed that there was regional heterogeneity in the impact of environmental regulation on GTFP based on the significance of the nonlinear impact in the eastern and western regions. The influence was not obvious in the central region.

The main contributions of this study to the literature are as follows. Firstly, we incorporated environmental regulation, governance transformation, and GTFP into a unified analytical framework in which the internal relationship between the three variables was investigated. Therefore, the research conclusions provide a more comprehensive understanding of the inherent mechanisms of the green growth effects on environment regulation. Secondly, we introduced resource exhaustion, environmental pollution, and economic growth into a productivity evaluation system and estimated the GTFP of China’s 30 provinces by using the Global Malmquist-Luenberger (GML) index based on the directional distance function. This enabled us to inherit the advantage of traditional productivity indexes, e.g., the Malmquist-Luenberger index, while alleviating the infeasibility problem and providing a more comprehensive calculation process. Thirdly, the linear term and quadratic term of environmental regulation were both introduced into the econometric model to explore the possible nonlinear relationship between environmental regulation and GTFP. In addition, to provide a sound theoretical basis for formulating differentiated environmental policy systems, we further analyzed the regional heterogeneous effects of environmental regulation on GTFP by dividing the balanced panel into three subsamples, namely, the eastern region, the central region, and the western region. Fourthly, the interaction effect of environmental regulation and governance transformation was also added into the empirical model, testing the moderating effect of governance transformation on the relationship between environmental regulation and GTFP. Finally, we empirically investigated the impact of environmental regulation and governance transformation on China’s provincial GTFP by using a dynamic panel model and system-generalized methods of moment (SYS-GMM) to overcome the possible endogenous problem.

The remainder of this paper is organized as follows. Section 2 briefly reviews the relevant literature and proposes three theoretical hypotheses. Section 3 describes the data sources and methods used in the empirical analysis. Section 4 discusses the empirical estimation results. Section 5 concludes the study and highlights several policy implications.

## 2. Literature Review and Research Hypotheses

### 2.1. Literature Review

When it comes to environmental regulation, governance transformation, and GTFP, previous studies have focused on the relationship between environmental regulation and GTFP, the relationship between governance transformation and GTFP, and the realization of the conditions of the Porter hypothesis. Therefore, in this section, we also review the related literature from these three aspects.

#### 2.1.1. The Relationship between Environmental Regulation and GTFP

With the increasing attention to the quality of the ecological environment, many scholars have adopted various methods to explore the impact of environmental regulation on GTFP [9,26,27,28,29]. However, the existing literature has not yet reached a consensus on the relationship between environmental regulation and GTFP [13]. Some studies hold the view that the essence of environmental regulation is to internalize the negative externalities of pollution, for it will inevitably bring additional costs to enterprises. Therefore, environmental regulation will inhibit the improvement of productivity [30,31]. Hancevic [12] claimed that environmental regulation will restrain the improvement of productivity owing to increases in pollution control costs. Zhang and Jiang [32] investigated the effect of the environmental policy on a firm’s green productivity by using Chinese coal-fired power plant data from 2005 to 2010 and found that stringent environmental regulation will damage green productivity growth. Cai and Ye [33] discussed the impact of the enforcement of China’s new environmental protection law on enterprises’ TFP by using DID model and proved that environmental regulation can give rise to a decline in enterprises’ TFP due to financial constraints. This conclusion is also supported by Du et al. [15] who indicated that environmental regulation would restrain green technology innovation. Conversely, some scholars believe that strict and well-designed environmental regulation can stimulate corporate innovation activities aimed at reducing compliance costs, enhance enterprises’ competitiveness, and promoting their productivity [34]. Lanoie et al. [35], using survey data from seven OECD countries in 2003, examined the effect of environmental regulation on productivity. They confirmed the weak Porter hypothesis and concluded that environmental regulation could promote TFP growth. Wang and Shen [36] focused on the effect of environmental regulation on the environmental productivity of China’s industries over the period from 2000 to 2012 and found that environmental regulation and environmental productivity were positively correlated, which, to a certain extent, validates the Porter hypothesis. Based on the panel data of the OECD countries’ industrial sectors between 2004 and 2009, Wang et al. [13] discussed the effect of environmental policy stringency on green productivity growth and confirmed that environmental regulation had a positive impact on green productivity growth. Zhang [14] adopted the dynamic panel model to explore the impact of environmental regulation on the green productivity of Chinese manufacturing firms and found that environmental regulation facilitated GTFP, especially in state-owned enterprises. In addition, some people have claimed that the impact of environmental regulation on GTFP is uncertain, for the compliance cost effects and the innovation compensation effects exist simultaneously. Based on the panel data of China’s carbon-intensive industries from 2000 to 2014, Zhao et al. [37] found that there was an inverted U-shaped relationship between environmental regulation and GTFP, demonstrating the inexistence of the Porter hypothesis in the long term. Qiu et al. [38] employed the FGLS model and the dynamic GMM model to analyze the effect of environmental regulation on the GTFP of China’s industrial sectors and verified a nonlinear U-shaped curve relationship between environmental regulation and GTFP. This result is consistent with that of Shen et al. [39] who found that the effect of environmental regulation on GTFP exhibited a significant U-shaped relationship from a provincial perspective. However, none of the above literature has examined the impact of environmental regulation on China’s provincial GTFP, which is of great significance for formulating differentiated environmental regulation policies to promote GTFP growth.

#### 2.1.2. The Relationship between Governance Transformation and GTFP

Regarding the relationship between governance transformation and GTFP, most firm-level evidence showed that market-oriented governance transformation, as an important institutional arrangement, can significantly facilitate GTFP. From the perspective of the influence mechanisms, three research hypotheses have been formed. The first hypothesis is the transaction cost effects. A well-developed marketization mechanism can effectively reduce the transaction costs and the investment risk of enterprises, which contributes to the enhancement of the profitability and production efficiency [21,40,41]. Zhang and Liu [20] argued that enterprises located in more developed market systems were highly related to more bank loans and other financial intermediaries, because efficient financing channels can fully meet the amount of capital investment required by enterprises to achieve the targeted GTFP. Therefore, market-oriented reforms raise the enterprises’ return on capital and investment, as well as improving GTFP [19,22]. The second hypothesis is the resource allocation effects. It is widely accepted that market mechanisms can directly enhance the operational flexibility of enterprises through the rebound effect, which can help to decrease the originally expected inputs, as well as increasing outputs [42]. Moreover, high-level market systems can improve the allocation efficiency of resources among and within enterprises and can boost the productivity of enterprises [24]. Bin et al. [23] analyzed the effect of the within-industry allocation efficiency on firm productivities and argued that the improvement of the resource allocation efficiency had a strongly positive influence on TFP growth. This conclusion has been confirmed by Lin and Chen [10] who found that factor market distortion inhibited China’s GTFP growth. The third hypothesis is technological innovation effects. Some previous studies found that GTFP growth was significantly positively correlated with innovation and that well-developed market systems stimulated the innovative activities of enterprises aimed at obtaining high profits, which contributed to the promotion of the enhancement of production efficiency [13,43,44]. Audretsch and Belitski [45] adopted firm-level unbalanced panel data to examine the effect of R&D on productivity and verified that the effort of innovation had a positive effect on green productivity by promoting technology transfer in a well-developed marketization environment. Zhang and Vigne [46] found that innovation efficiencies had a positive and significant impact on GTFP because of the benefits of market-oriented reforms. However, few scholars have empirically tested the impact of governance transformation on China’s GTFP from the provincial level perspective, especially the impact of regional heterogeneity on the relationship between governance transformation and GTFP, implying that geographic location is an important factor affecting the growth effect of governance transformation.

#### 2.1.3. The Realization Conditions of the Porter Hypothesis

The Porter hypothesis posits that well-designed environmental regulation can stimulate enterprises to carry out technological innovation and engender innovation compensation effects which help to improve the competitiveness of enterprises and their productivity [34,47]. Therefore, taking advantage of the innovation effects of environmental regulation is the key to achieve a win-win situation between environmental protection and economic development [48]. Accordingly, the existing literature mainly discusses the realization of the conditions of the Porter hypothesis from the following two aspects. On the one hand, some scholars discussed the impact of the fiscal policy, foreign direct investment, financial development, industrial structures, and factor endowment on the Porter hypothesis from the macro-level perspective. For example, Song et al. [49] analyzed the compound effects of fiscal decentralization and environmental regulation on GTFP in the Yangtze River economic belt and found that appropriate fiscal decentralization can contribute to the realization of the Porter hypothesis. Qiu et al. [38] explored the relationship between environmental regulation, FDI, and industrial GTFP, based on Chinese provincial panel data, and confirmed that FDI can accelerate the realization of the Porter hypothesis through the channel of the technology spillover effect. Lv et al. [50] examined the impact of financial development on green innovation under environmental regulation and confirmed that the financial structure was conducive to the realization of the Porter hypothesis, while the financial scale and financial efficiency had a negative effect on green development. Wang and Shen [36] investigated the relationship between industry structure changes and environmental productivity by using the Lema index and verified that industrial agglomeration had a significant positive effect on the innovation effects of environmental regulation. Xie et al. [51] employed a panel threshold model to examine the relationship among environmental regulation, human capital, and GTFP, and confirmed that the improvement of human capital structure significantly promoted the realization of the Porter hypothesis. On the other hand, some people discussed the impact of the enterprise ownership structure, the enterprise trade advantage, and enterprise resource misallocation on the Porter hypothesis from the microeconomic perspective. Peng et al. [25] confirmed that state-owned enterprises had lower productivity, on average, than non-state-owned enterprises, based on a large panel of data on Chinese industrial enterprises from 1998 to 2007, indicating that the impact of the ownership structure on the Porter hypothesis was very significant. Tang et al. [52] used a difference-in-difference framework to estimate the impact of export on productivity under environmental regulation and verified that an export advantage contributed to the realization of the Porter hypothesis. Based on the panel data of Chinese listed companies, Cai and Ye [33] confirmed that bank credit misallocation inhibited the realization of the Porter hypothesis. The conclusion is consistent with Lin and Chen [10] who found that factor market distortion had a negative influence on China’s GTFP growth. Most notably, the reform of the economic system was an important factor affecting the realization of the Porter hypothesis. To date, few scholars have investigated whether an increase in the environmental regulation intensity will affect China’s GTFP growth through the moderating effect of governance transformation.

### 2.2. Research Hypotheses

Based on the existing research and the reality of China’s industrial economic development, in this section we analyze the influence mechanisms of environmental regulation and governance transformation on GTFP systematically, and we put forward three research hypotheses to be tested.

#### 2.2.1. The Theoretical Mechanism of Environmental Regulation on GTFP

From the previous literature, it should be noted that the impact of environmental regulation on GTFP mainly depends on the comprehensive effects of an innovation decline and a value promotion [13]. To be specific, the decline in innovation is due to the decrease in innovation resources, which are mainly encroached by environmental regulation costs. The improvement of value is due to the contribution of the green process innovation, which can be spawned by environmental regulation. On the whole, the previous studies have greatly deepened our understanding of the effect of environmental regulation on GTFP; however, what we need to highlight is that the great majority of these studies ignored the dynamic effects of environmental regulation when analyzing its role. Specifically, when environmental supervision is less strict, the implementation of environmental regulation not only increases the pollution control costs but also stimulates extensive production and restrains green economic efficiency, which are referred to as the “cost effect” and the “crowding-out effect”, respectively [34]. However, with the improvement of environmental regulation stringencies, enterprises must continue to increase their environmental investment to carry out green technology innovations. This gradually compensates for the compliance costs and contributes to green productivity in the long run, which is called the “innovation compensation effect” [38,53]. Thus, environmental regulation will weaken the enterprises’ competitiveness in the early stages, but it can stimulate innovation and promote productivity over the long term. Hence, the following hypothesis is proposed:

**Hypothesis** **1** **(H1).** *Environmental regulation may inhibit GTFP in the short term and can promote GTFP in the long term and there is a U-shaped relationship between environmental regulation and GTFP.*

#### 2.2.2. The Theoretical Mechanism of Governance Transformation on GTFP

Theoretically, governance transformation reflects the persistent improvement of the degree of marketization in a country or a region. The socialist governance transformation can be categorized into two models: the Soviet model, which strongly argues for shock therapy, and the Chinese model, which advocates for a gradual reform. Compared with the Soviet model, the Chinese model can better cultivate a unified and open market economy and can create a fair and orderly institutional environment for private enterprises. In addition, it can improve the innovation incentive mechanisms, optimize resource allocation efficiencies, and promote enterprise productivity. From the previous literature, in order to clarify how market-oriented governance transformation affects GTFP, the key is to find out the way towards the improvement of GTFP. From the perspective of enterprises, the sources of GTFP growth are determined by two fundamental forces [23,54]. The first is the improvement of the corporate internal production efficiency caused by technological progress, which stems from R&D investment, technology introduction, a deepening of division of labor, the enhancement of the management level, and the optimal allocation of internal resources [10]. The second is the improvement of resource allocation efficiencies among enterprises, specifically where production factors flow from low productivity enterprises to high productivity enterprises, including the creative destruction process, in which low productivity enterprises continue to withdraw from the market and high productivity enterprises continue to enter the market [21]. Hence, by relying on the specialized division of labor and the adoption of advanced green technologies, market-oriented governance transformation can not only promote the enhancement of the internal production efficiency of enterprises, but it can also effectively raise the efficiency of resource allocations among enterprises, which can improve the overall green productivity of the whole society. Therefore, the following hypothesis is formulated:

**Hypothesis** **2** **(H2).** *Governance transformation can promote GTFP improvement.*

#### 2.2.3. The Theoretical Mechanism of Governance Transformation on the Porter Hypothesis

The previous research showed that the market-oriented governance transformation was helpful for improving the market competition and promoting corporate technological innovation [20]. Whether the governance transformation has a compound effect on the Porter hypothesis depends largely on the nature of the innovation effect of the governance transformation. Ettlie et al. [55] demonstrated that technological innovation can be divided into incremental innovation and radical innovation. Radical innovation refers to a kind of innovation that can produce a significant impact on market rules, the competition situation, and industrial layout, and may even lead to an industrial reshuffle [56,57]. Therefore, strengthening radical innovations and promoting the transformation of corporate technological innovations from incremental innovations to radical innovations are not only an important way for enterprises to break through technological bottlenecks and achieve an innovation catch-up, but they are also the key to the compound effect of governance transformation on the Porter hypothesis [45,58].

From the perspective of corporate governance, state-owned enterprises still contain more administrative governance factors because the reform is not in place, while private enterprises have more economic governance factors. Therefore, the innovation characteristics of enterprises with different ownerships will show differences due to different governance mechanisms. That is, state-owned enterprises prefer incremental innovations, while private enterprises hope to obtain radical innovations for subverting the existing market structure. Specifically, in the early stages of environmental regulation, radical innovation is not easy to realize. State-owned enterprises can achieve incremental innovations because they can obtain many subsidies from the government. Thus, the green competitiveness of state-owned enterprises is stronger than that of private enterprises. In the later stages of environmental regulation, the radical innovations of private enterprises are gradually realized, while the radical innovations of state-owned enterprises are slow due to the difficulty of governance transformation. At this time, the green competitiveness of private enterprises will exceed that of state-owned enterprises. Therefore, the governance transformation can accelerate the realization of radical innovations and can promote the improvement of GTFP. Based on this, the following hypothesis is proposed:

**Hypothesis** **3** **(H3).** *Governance transformation can accelerate the realization of the Porter hypothesis; namely, governance transformation contributes to the achievement of the improvement of environmental regulation on GTFP.*

**Figure 2:**
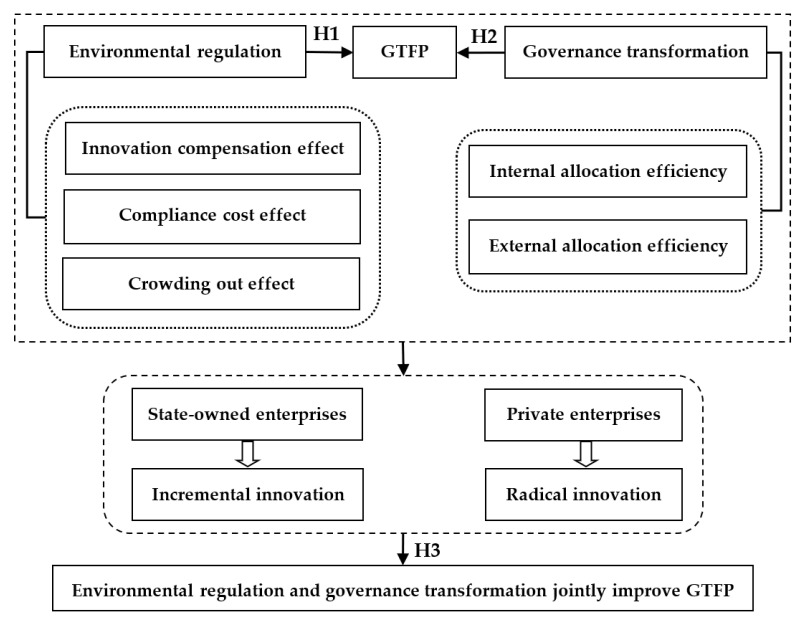
The theoretical mechanism diagrams of hypotheses.

In summary, the theoretical mechanisms of the hypotheses constructed in this paper can be summarized in Figure 2. As can be seen in Figure 2, environmental regulation will affect GTFP through three channels: the innovation compensation effect, the compliance cost effect, and the crowding out effect. Thus, the research Hypothesis 1 (H1) is obtained. Meanwhile, governance transformation can not only improve the internal production efficiency of enterprises, but it can also enhance the allocation efficiency among enterprises, which can promote GTFP. Therefore, the research Hypothesis 2 (H2) is formed. In addition, the innovation characteristics of enterprises with different ownerships will exhibit significant differences due to different governance mechanisms; namely, state-owned enterprises prefer incremental innovation, while private enterprises hope to obtain radical innovation for subverting the existing market structure. Accordingly, we can put forward the research Hypothesis 3 (H3).

## 3. Econometric Methodology and Data

### 3.1. Econometric Model Specification

Under the new growth theory, economic growth is mainly promoted by capital, labor, and technological progress. In addition, previous studies have shown that environmental regulation, the optimization of resource allocation, and R&D investment can give rise to technological progress. Similarly, FDI and export, can bring about technology spillovers under the condition of an open economy [8,59]. Accordingly, the production function can be described as follows:

(1) $$Y(y_{desire},y_{undesire})=A(er,gt,rd,fdi,ex,t)\cdot F(K,L)$$

In Equation (1), *Y* represents the total output, consisting of the desired output and the undesired output; *A* stands for the green total factor productivity (GTFP) considering undesired output; *er* denotes environmental regulation; and *gt*, *rd*, *fdi*, and *ex* represent governance transformation, R&D investment, foreign direct investment, and export, respectively. *K* and *L* indicate capital and labor, respectively. In addition, *A* indicates the Hicks neutral technology progress function. For simplicity, in reference to the study by Zhang [22], we assume that *A* in Equation (1) includes a variety of components, as follows:

(2) $$A(er,gt,rd,fdi,ex,t)=A_{i0}e^{\lambda_{i}t}er_{it}^{\alpha_{i}}gt_{it}^{\beta_{i}}rd_{it}^{\gamma_{i}}fdi_{it}^{\delta_{i}}ex_{it}^{\tau_{i}}$$

By substituting Equation (2) into Equation (1), we get the following equation:

(3) $$Y_{it}(y_{desire_{it}},y_{undesire_{it}})=A_{i0}e^{\lambda_{i}t}er_{it}^{\alpha_{i}}gt_{it}^{\beta_{i}}rd_{it}^{\gamma_{i}}fdi_{it}^{\delta_{i}}ex_{it}^{\tau_{i}}\cdot F(K_{it},L_{it})$$

where *i* and *t* denote the region and time, respectively; *A_i0_* represents the initial productivity level; *λ_i_t* reflects exogenous productivity changes; and *α_i_*, *β_i_*, *γ_i_*, *δ_i_*, and *τ_i_* represent the parameters of environmental regulation, governance transformation, R&D investment, foreign direct investment, and export trade on GTFP, respectively.

In terms of the definition of GTFP, we divide the two sides of Equation (3) by using *F*(*K_it_*,*L_it_*) to obtain the following formula:

(4) $$GTFP_{it}=\frac{Y_{it}(y_{desire_{it}},y_{undesire_{it}})}{F(K_{it},L_{it})}=A_{i0}e^{\lambda_{i}t}er_{it}^{\alpha_{i}}gt_{it}^{\beta_{i}}rd_{it}^{\gamma_{i}}fdi_{it}^{\delta_{i}}ex_{it}^{\tau_{i}}$$

By taking the natural logarithm of both sides of Formula (4), we obtain the theoretical framework describing the impact factors of GTFP, as follows:

(5) $$\ln GTFP_{it}=\ln A_{i0}+\lambda_{i}t+\alpha_{i}\ln er_{it}+\beta_{i}\ln gt_{it}+\gamma_{i}\ln rd_{it}+\delta_{i}\ln fd_{it}+\tau_{i}\ln ex_{it}$$

To correctly explore the effect of environmental regulation, governance transformation, and their interaction terms on GTFP, based on the above analysis framework, the linear term and the quadratic term of environmental regulation are both introduced into the econometric model to investigate the possible nonlinear relationship between environmental regulation and GTFP. Since the current productivity growth may be affected by past productivity, the first-order lag term of GTFP is also included in the model as an explanatory variable to examine the dynamic cumulative effect of GTFP growth. Meanwhile, the estimation method, the system generalized method of moments (GMM), is used in this paper mainly because the system GMM can effectively overcome the endogenous problem by introducing instrumental variables. Based on the above considerations, the dynamic panel regression model constructed in this study is as follows:

(6) $$\ln GTFP_{it}=\alpha_{0}+\alpha_{1}\ln GTFP_{it-1}+\alpha_{2}\ln ER_{it}+\alpha_{3}{\left(\ln ER_{it}\right)}^{2}+\alpha_{4}\ln GT_{it}+\beta X_{it}+V_{i}+\varepsilon_{it}$$

where *i* and *t* represent the province and year, respectively (*i* = 1, 2, 3,…, 30, *t* = 2003, 2004, …, 2017); *V_i_* indicates the provincial fixed effect; ε*_it_* is the random error term; and *GTFP_it_* denotes the green total factor productivity for each province; *α_1_* is a hysteresis multiplier, indicating the effect of the last period of GTFP on the current GTFP; *ER_it_* denotes environmental regulation; *GT_it_* represents governance transformation; and *X_it_* is the control variables vector, including R&D investment, export trade, foreign direct investment, and the factor endowment structure, respectively. To be specific, the coefficient of the quadratic term of ER illustrates the nonlinear effect of environmental regulation on GTFP. When the estimated value of parameter *α_3_* is significantly more than 0, Hypothesis 1 is confirmed. Simultaneously, when the result of the parameter *α_4_* is significantly greater than 0, Hypothesis 2 is confirmed. In addition, the interaction term of *ER* and *GT* is included in the model to verify Hypothesis 3. The dynamic panel regression model with the cross-term is established as follows:

(7) $$\begin{array}{l} \ln GTFP_{it}=\alpha_{0}+\alpha_{1}\ln GTFP_{it-1}+\alpha_{2}\ln ER_{it}+\alpha_{3}{\left(\ln ER_{it}\right)}^{2}+\alpha_{4}\ln GT_{it}\\ +\alpha_{5}\ln ER_{it}\times \ln GT_{it}+\beta X_{it}+V_{i}+\varepsilon_{it} \end{array}$$

In Equation (7), ln*ER_it_*×ln*GT_it_* indicates the cross-term of environmental regulation and governance transformation. When the estimated result of the coefficient of the interaction term is significantly greater than 0, Hypothesis 3 is confirmed.

### 3.2. Variable Selection and Description

#### 3.2.1. Dependent Variable

The dependent variable used here is green total factor productivity (GTFP). In general, productivity indexes used in prior studies focused on measuring marketable outputs relative to the paid factors of production. However, the generation of by-products, such as environmental pollutants, was not taken into consideration. In view of this, Chung et al. [60] proposed the Malmquist-Luenberger index, based on the directional distance function, which extends the original Malmquist index to account for undesired outputs, such as industrial sulfur dioxide, industrial smoke and dust, and industrial sewage. Therefore, to overcome the drawback of lacking environmental factors in the traditional productivity index, and to inherit the advantages of the Malmquist-Luenberger index following Chung et al. [60] and Oh [61], this paper uses the global Malmquist-Luenberger index (GML), based on the directional distance function, to measure the GTFP growth for each province in China. The GML index used in this study is defined as follows:

(8) $$GML^{t,t+1}(x^{t},y^{t},b^{t},x^{t+1},y^{t+1},b^{t+1})=\frac{1+D^{G}(x^{t},y^{t},b^{t})}{1+D^{G}(x^{t+1},y^{t+1},b^{t+1})}$$

In Equation (8), *D^G^*(•) represents the directional distance function; *x^t^* represents the input vector constructed by *N* kind of input factors; *y^t^* represents the desirable output vector constructed by *M* kind of desired outputs; and *b^t^* represents the undesirable output vector constructed by *I* kind of undesired outputs. Specifically, the input factors include: (1) capital input, where the capital stock of each province is calculated by employing the perpetual inventory method [9]; (2) labor input, measured by the annual average number of people employed for each province; and (3) energy consumption, expressed by the provincial primary energy consumption, which is converted to standard coal. In addition, the output factors include: (1) the desirable output, denoted by gross industrial output; and (2) the undesirable output, measured by a variety of environmental pollutants, including industrial sewage emissions, industrial sulfur dioxide, and carbon dioxide emissions.

#### 3.2.2. Core Independent Variables

- Environmental regulation (ER). According to the Porter hypothesis, reasonable environmental regulation can effectively stimulate enterprise enthusiasm for green innovation, promote pollution emissions reduction, and improve green productivity. In previous studies, there were no clear and unified criteria to represent environmental regulation, which can mainly be measured by pollutant emission reductions, environmental pollution control investments, pollution reduction expenditures, regulatory enforcement stringencies, and pollution sewage charges [4,39,62,63]. For the consideration of data integrity and availability, this paper selects the ratio of the total investment of provincial industrial pollution control to the gross industrial output as a proxy variable for the provincial environmental regulation level. Usually, the higher the proportion of the investment, the greater the environmental regulation intensity, and the better the effect of regional green development. In addition, we also select the ratio of the total investment of industrial pollution control to the operating cost of industrial enterprises, as a substitute variable for the robustness analysis.
- Governance transformation (GT). China’s corporate governance, which is deeply rooted in the transitional economy, is gradually transforming from administrative governance to economic governance. In essence, governance transformation reflects the persistent improvement of the degree of marketization for a region. Generally speaking, private enterprises have better economic governance, while state-owned enterprises have stronger administrative governance in China. Therefore, the governance transformation, which is measured by the ratio of the main business income of private industrial enterprises to the sum of the main business income of state-owned and private-owned industrial enterprises for each region, is selected here. The larger the ratio of governance transformation, the higher the degree of marketization, and the better the resource allocation and productivity of enterprises.

#### 3.2.3. Control Variables

To alleviate the endogenous problem caused by the omitted variables, and to improve the estimation accuracy, a series of control variables are selected in the model by referring to the previous literature. The variables included are as follows:

- R&D investment (RD). Technological innovation is the important driving force for the improvement of regional green total factor productivity, which is conducive to the optimized resource allocation of enterprises, the enhancement of the product quality, and the reduction of pollution emissions [27,48,64]. In this paper, R&D investment is measured by the ratio of R&D internal expenditure to regional industrial GDP.
- Export trade dependence (EX). Previous studies indicate that export is highly correlated with regional green development [9]. China has grown into the world’s largest exporter over the past ten years. Export not only helps to expand foreign demand, to finance R&D, and to stimulate green innovation, but it may also increase local pollution emissions. To investigate the effect of export expansions on regional green development, we choose the ratio of the total export volume to the provincial GDP as agent indicators for the analysis [49,65].
- Foreign direct investment (FDI). As a main channel for international industrial linkages and technology spillovers, FDI can not only change domestic capital markets, but it can also promote the improvement of GTFP [38]. Thus, foreign direct investment, which is represented by the proportion of annual foreign investment actually utilized in GDP, is regarded as a control variable in this study.
- Factor endowment structure (K/L). China’s industry is gradually transforming from an extensive model to an intensive model. Compared to labor-intensive industries, capital-intensive industries usually use relatively advanced technology and equipment, which is beneficial to the improvement of resource efficiency and the green transformation of industry [5,66]. Therefore, the ratio of capital to labor is used to represent the factor endowment structure for each region.

### 3.3. Data Sources and Variable Descriptive Statistics

Considering the data availability and the actual needs of the research, this paper selected the Chinese provincial panel data between 2003 and 2017 to empirically investigate the impact of environmental regulation on green total factor productivity. To be specific, our sample covers 30 provinces, municipalities, and autonomous regions in mainland China from 2003 to 2017. Due to a shortage of the portion of required data, Hong Kong, Macao, Taiwan, and Tibet are excluded here. The sample dataset used in the study was mainly derived from the China Statistical Yearbook, the China Energy Statistical Yearbook, the China Environment Statistical Yearbook, the China Industrial Statistical Yearbook, the China Industrial Economic Statistical Yearbook, the China Science and Technology Statistical Yearbook, and each province’s Provincial Statistical Yearbook for each sample year. The methods of moving the averages and interpolations were applied to supplement the missing data in some years and regions. In total, a balanced sample set of 450 observations was created for each region during this 15-year period. Furthermore, all variable values in the study were transformed into logarithmic forms to reduce heteroscedasticity. In order to eliminate the price effect, we deflated all the nominal variables in this study into real variables, by a GDP deflator, into the 2003 constant price. The descriptive statistics of all aforementioned variables are summarized in Table 1 below.

## 4. Empirical Results Analysis

### 4.1. Unit Root Test and Multicollinearity

In order to eliminate the spurious regression problem and to ensure that the estimation results are accurate and reliable, the stationarity test of all variables was implemented before the regression analysis. The test methods consisted of LLC, IPS, Fisher-ADF, and Fisher-PP tests. As shown in Table 2, the test results showed that almost all of the variables can pass more than three significance tests simultaneously, indicating that the raw data sequence of each variable was stationary. In addition, the variance inflation factor (VIF) was used to test the multicollinearity problem. The results of the VIF test indicated that the VIF values were all less than 10 and ranged from 1.24 to 2.22, showing that there was no multicollinearity among the variables. Therefore, the regression analysis was performed.

### 4.2. The Spatial-Temporal Dynamic Evolution of Regional GTFP in China

**Figure 3:**
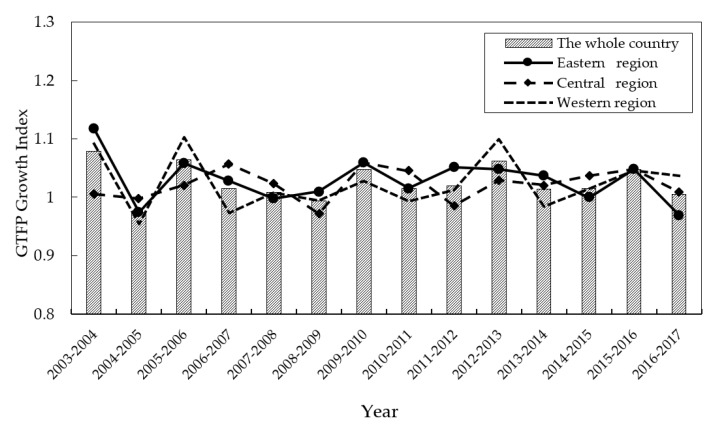
Trends of regional average GTFP growth index in China from 2003 to 2017.

**Figure 4:**
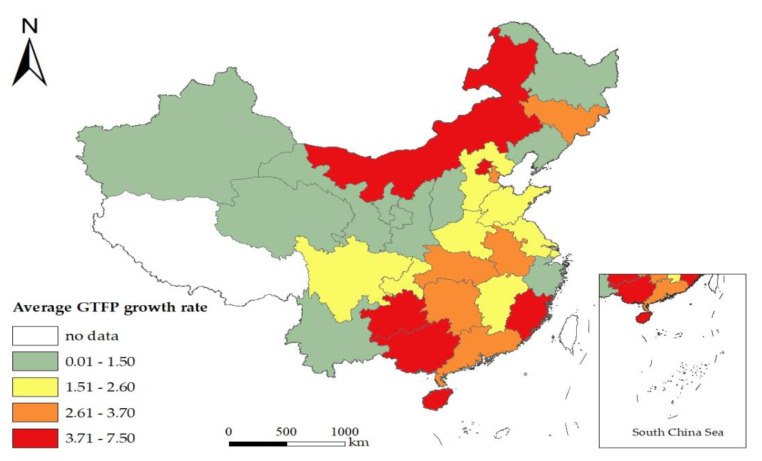
Spatial distribution of regional average GTFP growth rate in China from 2003 to 2017.

Based on the panel data of 30 provinces in China over the period from 2003 to 2017, this study adopted a directional distance function and the GML index to measure the regional GTFP growth, as shown in Figure 3 and Figure 4.

Figure 3 shows the overall trend of China’s GTFP from 2003 to 2017. The average annual growth rate of GTFP dropped from 7.8375 in 2003 to 0.4687 in 2017, indicating that the GTFP presented an overall downward trend during these 15 years. To be specific, the GTFP declined from 2003 to 2007, followed by a slight rise from 2008 to 2010 and further fluctuates until 2013, although these variation features are not very obvious. During the 11th and 12th Five-Year Plans, the government enhanced the governance of energy conservation and the emission reduction of enterprises, gradually strengthening the responsibility of local governments to control their environmental pollution. Due to the long-term dependence on the extensive economic development model and the occurrence of the financial crisis in 2008, China has implemented a series of stimulus programs to promote infrastructure investment and heavy industries. As a result, the extensive production conditions caused by resource consumption and environmental pollution have not substantially improved. In 2013, the average growth rate of China’s GTFP rose to 6.1834, illustrating that environmental supervision has brought about some positive effects. However, since 2013, when China’s economy entered a new normal period, the pressure of the growth slowdown caused a decline in GTFP, which decreased to 0.4687 in 2017.

In terms of changes in different regions, the levels of economic development in the eastern, central, and western regions were different, and the changes in GTFP varied accordingly (see Figure 3 and Figure 4). Specifically, between 2008 and 2014, the eastern region experienced rapid economic growth and the contribution of GTFP increased from 0.9425 in 2008 to 3.662 in 2014, after which it began to decline rapidly. From 2006 to 2012, the GTFP growth in the western region fluctuated little. Since 2013, the GTFP growth rate in the western region has shown a downward trend, declining from 9.9725 in 2013 to 3.7044 in 2017. From 2005 to 2015, the GTFP growth in the central region fluctuated little, showing the steady contribution of GTFP to economic development. However, the GTFP growth rate rose to 4.7981 in 2016 and then began to decline rapidly, falling to 0.8975 in 2017.

Since the reform and expansion, the central region has given priority to the development of heavy industry to achieve rapid economic growth. Due to the lack of physical capital, human capital, and advanced technology, the effect of the industrial policy on economic growth was restricted to a great extent. Therefore, the local government relaxed the punishment for resource damage and environmental pollution, trying to sacrifice the environment in exchange for the rapid economic growth. However, this extensive development model is doomed to be unsustainable. Although environmental governance was strengthened in 2015, the region is still unable to get out of the development pattern characterized by high pollution and high energy consumption.

The western region is rich in natural resources and energy and has a strong economic development potential. With the implementation of the Western Development Strategy and the latecomer advantage, some developed areas have witnessed rapid economic growth, which will inevitably accelerate energy consumption and environmental pollutants emission, resulting in a poorer performance of GTFP long-term.

### 4.3. The Analysis of Baseline Empirical Results

The purpose of this paper is mainly to test whether the nonlinear U-shaped relationship between environmental regulation and GTFP exists or not. Furthermore, considering the background of the Chinese market-oriented reform, governance transformation is also introduced into the model to investigate its impact on GTFP. Specifically, we first used the feasible generalized least squares (FGLS) method, the fixed effect (FE) method, and the random effect (RE) method to explore the effects of environmental regulation and governance transformation on GTFP, respectively. The estimation results are presented in columns 1–6 of Table 3. As we expected above, the coefficients of the quadratic term of environmental regulation were positive, and so were the coefficients of governance transformation. However, the fitting degrees of these six models were all less than 0.4, indicating that these three estimation methods were not able to explain causality effectively. In addition, if the explanatory variables are endogenous, the FGLS method, the FE method, and the RE method may lead to inconsistencies in parameter estimations. Therefore, in order to effectively overcome the endogeneity problem, we used the system generalized method of moments (SYS-GMM) to estimate the models by introducing instrumental variables. The difference generalized method of moments (DIFF-GMM) was also used to ensure that the regression results were robust. The estimation results of the DIFF-GMM method and SYS-GMM method are shown in columns 7 and 8 and columns 9 and 10 of Table 3, respectively. It should be noted that the AR tests, which are used to test the autocorrelation of the residual term, showed that there was a first-order autocorrelation but there was no evidence of a second-order autocorrelation. Meanwhile, the Hansen over-identification tests, which are usually adopted to examine the validity of the instrumental variables, indicated that the null hypothesis cannot be rejected; namely, the instrumental variables were jointly effective. Therefore, the specifications of the dynamic panel data models in this study are reasonable.

Firstly, the coefficients of the first-order lag term of GTFP were all significantly positive at the 1% level in Models (7)–(10), showing that the growth of GTFP is a dynamic accumulation process with significantly positive feedback and path dependency. The SYS-GMM estimation results of the environmental regulation showed that the coefficients of the linear term of environmental regulation were all significantly negative, while the coefficients of the quadratic term were significantly positive, indicating that there is a nonlinear U-shaped relationship between environmental regulation and GTFP. As a comparison, the DIFF-GMM estimation results showed that the regression results were robust. Therefore, Hypothesis 1 (H1) is verified. The feasible explanation for this nonlinear relationship is that when environmental supervision is less strict, the pollution control costs and the extensive production are higher, and the green economic efficiency is lower. However, with the improvement of environmental regulation stringencies, enterprises must continue to increase environmental investment to carry out green technology innovation, which will gradually compensate for the compliance costs and contribute to green productivity in the long term. Furthermore, taking Model (10) as an example, the inflection point value of environmental regulation is 3.065, which is greater than the mean level of 2.259, shown in Table 1, indicating that the intensity of environmental regulation in China is still located on the left side of the U-shaped curve, meaning that the promotional effect of environmental regulation on GTFP has not been achieved fully.

Secondly, the coefficients of governance transformation were all positive and significant in Model (8) and Model (10), showing that governance transformation can promote the improvement of GTFP. Hypothesis 2 (H2) is, thus, verified. The possible explanation for this is that with the establishment and improvement of the socialist market economic system, the market-oriented governance transformation plays an increasingly important role in the efficiency of the resource allocation among enterprises. Therefore, resources will flow into high efficiency enterprises over time, which contributes to the enhancement of the internal production efficiency of enterprises by the specialized division of labor and the adoption of advanced green technology. This will then improve the overall green productivity of the whole society in the long term.

Finally, among the control variables, the coefficients of R&D investment were significantly positive, showing that R&D investment can promote the growth of GTFP. The credible explanation for the result is that the increase in R&D investment is beneficial to the enhancement of the innovation capacity of enterprises through the promotion of technological progress, thus improving production efficiency. The coefficients of FDI were always positive but not significant, indicating that FDI cannot significantly improve GTFP. This is due to the fact that local governments pay more attention to the quantity of FDI but ignore the quality of FDI. As a result, the introduction of foreign-invested enterprises with a high production efficiency and green technology is insufficient, which affects the improvement of regional productivity. However, export trade dependence and the factor endowment structure have a negative impact on GTFP. The conceivable explanation is that the low-level export product quality and the unreasonable resource allocation models hinder the transformation of the extensive economic development characterized by high input, high emissions, and low efficiency and, thus, inhibit the growth of GTFP.

### 4.4. The Analysis of Regional Heterogeneity Results

Although the regression results of the full sample show a nonlinear U-shaped relationship between environmental regulation and GTFP, governance transformation can improve GTFP. Due to China’s vast territories and the differences in the level of economic development among regions, whether the benchmark regression results are still tenable in different regions is, therefore, unknown. In order to solve this problem, the study divided the full sample into three sub-samples according to geographical locations and economic characteristics, which are the eastern region, the central region, and the western region. Then, we used the SYS-GMM method to estimate three sub-samples. The results are presented in Table 4.

The regression results of Models (1)–(6) in Table 4 show that the effects of environmental regulation on GTFP have regional differences. Specifically, the U-shaped relationship between environmental regulation and GTFP was significant in the eastern and western regions, but it was uncertain in the central region. The reasons can be summarized into three aspects. Firstly, cities in the eastern region have established third-party pollution control mechanisms and have obtained remarkable achievements. Compared to the other regions, industrial enterprises in the eastern region are more adaptable to stricter environmental standards. Secondly, following the findings by Song et al. [49], government subsidies will impair the positive effect of environmental regulation on technological innovation. In the central region, the R&D activities of environmental control are heavily dependent on government investments, so that the enterprise benefits from technological innovation investment are not enough to compensate for the additional production costs caused by environmental regulation. In addition, in order to stimulate economic development, local governments compete to set lower environmental regulatory standards to attract investment projects. For this reason, environmental regulation policies become a mere formality. Thirdly, in the western region, owing to the relatively backward economic development level, many enterprises are able to receive more favorable policies under the national implementation of the Western Development Strategy. With the strengthening of environmental regulation, more green funds provided by local governments are used to support enterprises to carry out green technology innovations, which improves the green production efficiency in the long term.

Meanwhile, the regression coefficients of governance transformation in the eastern region, central region, and western region were 0.0202, 0.0621, and 0.0215, respectively, indicating that the promotional effect of governance transformation on GTFP in the central region is greater than that in the eastern region and western region. Because of the implementation of the Central Rise Policy, the state and the local governments have issued a series of policies to promote economic development, which stimulates the development of private enterprises in the central region. Under such circumstances, market-oriented governance transformation can effectively strengthen the efficiency of resource allocation among enterprises, which inspires the vitality of private enterprises with a high efficiency and promotes the realization of radical innovation, as well as improving the production efficiency in this region [44,46].

In addition, the influence of other control variables on GTFP also present regional differences. For example, the regression coefficients of export trade dependence in the eastern region were significantly negative, while those in the central and western regions were not significant. The regression coefficients of the factor endowment structure were significantly negative in the eastern and western regions, while those in the central region were not significant. The regression coefficients of FDI were significantly positive in the eastern region, while those in the central and western regions were not significant, mainly because the quality of FDI introduced in the eastern region is improved constantly and GTFP is also promoted. Moreover, the impact of R&D investment on GTFP in all regions was significantly positive, indicating that R&D investment contributes to the enhancement of enterprise innovation capacities, as well as improved production efficiency over time.

### 4.5. The Analysis of the Robustness Test Results

In order to ensure the reliability and validity of the baseline regression results, this paper used four ways to perform robustness tests. First, we used the dynamic panel threshold model to estimate the full sample. To be specific, according to the threshold variable, namely, the level of environmental regulation, the full sample was classified into a high group and a low group. The SYS-GMM method was then used to estimate the two groups simultaneously. Second, we adjusted the measurement pattern of environmental regulation. Specifically, the ratio of the total investment of industrial pollution control to the operating costs of industrial enterprises was selected as a substitute variable for environmental regulation. Then, the regression analysis was based on the adjusted full sample data. Third, the two-step SYS-GMM method was used to estimate the full sample. Compared with the one-step SYS-GMM, the two-step SYS-GMM can improve the estimation efficiency, as the weight matrix of the instrumental variables can be modified by the residual matrix of the one-step SYS-GMM. Fourth, we adjusted the sample interval for estimation. Specifically, the samples in 2003 and 2017 were excluded to eliminate the impact of the sample time selection on the estimation results. Thus, the provincial panel data from 2004 to 2016 were used for the re-estimation. The robustness test results are shown in Table 5.

Columns 1 and 2 in Table 5 report the results of the panel threshold regression model, indicating that there is a nonlinear U-shaped relationship between environmental regulation and GTFP. Specifically, when the level of environmental supervision is lower than the threshold value (3.15), environmental regulation inhibits the growth of GTFP. If the level of environmental supervision is greater than 3.15, environmental regulation can facilitate the improvement of GTFP. Furthermore, the coefficients of governance transformation were both significantly positive in the low and high groups. Columns 3 and 4 in Table 5 show the regression results when the measurement of environmental regulation is replaced, demonstrating that the coefficients of the quadratic term of environmental regulation were positive and significant, confirming the nonlinear U-shaped relationship between environmental regulation and GTFP. The coefficients of governance transformation were significantly positive. Columns 5 and 6 in Table 5 show the estimation results of the two-step SYS-GMM, illustrating that the relationship between environmental regulation and GTFP is nonlinear and U-shaped. The coefficients of governance transformation were also significantly positive. Columns 7 and 8 in Table 5 report the regression results of adjusting the sample interval, indicating that the nonlinear U-shaped relationship between environmental regulation and GTFP exists. The coefficients of governance transformation were positive. In addition, the regression results of the four robustness tests showed that the signs and significance of the coefficients of other control variables were consistent with the baseline empirical results. In conclusion, the robustness tests verified the existence of the nonlinear U-shaped relationship between environmental regulation and GTFP and that governance transformation can promote the growth of GTFP. Thereby, the baseline regression results of this study are robust and reliable.

### 4.6. Further Discussion

With the deepening of the socialist market economic reform, the role of the market mechanism in resource allocation has been increasingly strengthened. In essence, governance transformation reflects a market-oriented resource allocation reform, indicating that the governance model is changing from an administrative governance to an economic governance. Regarding the increasingly serious environmental problems, it is unknown if the market-oriented governance transformation can improve the effect of environmental regulation on GTFP and if the governance transformation can facilitate the realization of the Porter hypothesis and its influencing mechanisms. In order to solve these problems, the interaction term between governance transformation and environmental regulation was added into the model to investigate the influence of governance transformation on the Porter hypothesis. The regression results are shown in Table 6.

The estimation results reported in columns 1–8 show that the coefficients of the interaction term between governance transformation and environmental regulation are significantly positive, indicating that, with the improvement of the environmental regulation intensity, governance transformation plays an increasingly important role in promoting the growth of GTFP. In other words, governance transformation can accelerate the realization of the improvement effects of environmental regulation on GTFP. Hypothesis 3 (H3) is, thus, verified. The reason for the result is probably that as market plays an increasingly important role in resource allocation, the efficiency of resource allocation among enterprises can be improved, which contributes to the inspiration of the innovative enthusiasm of private enterprises in the face of stricter environmental regulations. Radical innovation can, therefore, be realized quickly, and this will promote productivity over the long term. In addition, the signs and significance of the coefficients of environmental regulation, governance transformation, and the other control variables are also consistent with the baseline regression results.

## 5. Conclusions

How to protect the ecological environment and promote high-quality economic development has gradually become a research hotspot in recent years. Under such circumstances, this paper uses the GML method to measure the GTFP of 30 provinces in China from 2003 to 2017 and investigates the impact and mechanisms of environmental regulation on GTFP by using a dynamic panel model and the SYS-GMM method. The main conclusions are drawn as follows. First, under the dual pressures of severe resource constraints and environmental protection, GTFP is an accurate and meaningful indicator of the measurement of the level of economic development. Second, there is a nonlinear U-shaped relationship between environmental regulation and GTFP, indicating that the Porter hypothesis is verified in China. Most notably, the intensity of environmental regulation is still located on the left side of the U-shaped curve, which means that the promotional effect of environmental regulation on GTFP has not yet been realized fully. Meanwhile, the nonlinear U-shaped relationship shows significant regional differences. It is the western region that presents the highest level of significance, followed by the eastern region, but it is insignificant in the central region. Third, governance transformation can significantly improve GTFP by promoting enterprise technological innovation. Fourth, governance transformation can accelerate the realization of the Porter hypothesis by inspiring the innovation enthusiasm of private enterprises. In other words, governance transformation contributes to the achievement of the realization of the improvement effects of environmental regulation on GTFP. Moreover, R&D investment can significantly improve GTFP, while the impacts of export trade dependence and the factor endowment structure on GTFP are negative and significant. The influence of FDI on GTFP is insignificant.

In order to uphold the guidance of green development and to promote high-quality economic development by preserving the ecological environment, based on the above conclusions, we propose the following policy recommendations.

Firstly, China should firmly hold the conviction that lucid waters and lush mountains are invaluable assets and should always adhere to an ecological priority and green development. Specifically, the Chinese government should closely monitor energy conservation, emission reductions, and ecological environment protection, as well as optimizing the ecological environment of factor allocation and enhancing the value creativity of factor resources, such as labor, capital, and energy. Meanwhile, the state and local governments should formulate a diversified green government performance appraisal system, especially for the official promotion evaluation mechanism, in which the weight of environmental indicators should be considered. Moreover, local governments should strengthen exchanges and cooperation in environmental governance and should establish inter-jurisdiction joint preventions and control coordination mechanisms to improve the ecological environment.

Secondly, the Chinese government should establish a long-term, institutionalized, and standardized environmental regulation policy system that is coordinated with regional economic development as soon as possible. To be specific, the central government should further strengthen the overall planning of environmental regulation across regions and establish a coordinated and unified environmental supervision system, such as setting up an inter-regional transfer payment system characterized by an ecological compensation mechanism. At the same time, the government should vigorously expand environmental regulation tools and comprehensively use legal, economic, technical, and administrative means to establish a fair and diverse environmental regulation tool system. In addition, the state and local governments should enhance the coordination between regional environmental policies and regional economic development policies, as well as implementing flexible and differentiated environmental regulation policies to improve the effectiveness of environmental regulation over time.

Thirdly, China should strive to create a fair and trustworthy market environment and give full play to the decisive role of the market in resource allocation. Specifically, the government should accelerate the market-oriented reform of the factor price in the capital market, labor market, and land market, as well as improving the transparency of market transactions to eliminate the factor price distortion. Meanwhile, the government should strengthen anti-monopoly policies, reduce entry constraints to monopoly industries, and promote the reform of the profit distribution systems of state-owned enterprises. In addition, the government should avoid excessive intervention in the economy, eliminating the differential treatment in credit supplies, interest rates, and market access, as well as improving the official administrative efficiency and public service levels.

Finally, in order to realize the green development of China’s economy, we should use the coordination effect of technological innovation, FDI, trade openness, and factor endowment. Specifically, (1) the state and local governments should increase their investment in R&D and should provide financial support for green technology innovation by setting up green development funds. In particular, the government should improve the incentive mechanisms for the commercialization of technological innovation achievements. (2) In order to fully exploit the spillover effect and pollution halo effects of FDI, the local governments should transform the FDI attraction mode from quantity to quality to attract high-quality technological FDI and green FDI based on local economic development circumstances. (3) China should continue to expand and optimize the foreign trade structure. It is important to accelerate the transition of the export trade from quantity to quality, such as reducing the proportion of export volume in low value-added and high pollution industries. Meanwhile, the import volume of new technologies, especially the green technologies, should be enlarged. (4) China should improve the factor market allocation and promote the free flow of capital, labor, and other factors. For example, the government should strengthen financial marketization reforms to effectively reduce the capital cost for enterprises and to deepen the Hukou (household registration) system reform to optimize labor allocations between urban and rural areas.

### Limitations and Outlook

This paper mainly explores the impact of environmental regulation on China’s regional green development from the governance transformation perspective. Although this study provides valuable insights, it still has certain limitations, which could also be directions for future research. First, owing to the difficulty in collecting relevant data, the sample period of this paper spanned from 2003 to 2017. Specifically, when calculating the GTFP growth of China’s 30 provinces, the data of these variables, such as gross industrial output, industrial sulfur dioxide, and industrial sewage emissions, were only updated to 2017. The index of environmental regulation, namely, the amount of industrial pollution control investment, lacks the data for 2018 in all provinces. If additional data had been available, the conclusions would be richer and more reliable. Future research can make progress on data expansion. Second, while this study investigates the nonlinear relationship between environmental regulation and GTFP in an empirical analysis, its driving mechanism and dynamic evolutionary paths have not been clearly explored, which is limited by sample data acquisition. Therefore, the follow-up studies can attempt to establish theoretical models (e.g., the dynamic stochastic general equilibrium model), which could be used to describe the dynamic transmission mechanisms of environmental regulation and governance transformation on GTFP, as well as providing a sound theoretical basis for the empirical results of this paper. Third, the dynamic panel GMM model used in this paper ignores the spatial effects of environmental regulation. However, due to the externality of environmental pollution, there are obvious imitation behaviors and strategic games in inter-regional environmental regulation, which inevitably engenders spatial spillover effects. In the future, we plan to divide environmental regulation into administrative environmental regulation and market-based environmental regulation. This will enable the construction of a dynamic spatial panel GMM model to investigate the direct and indirect effects of different types of environmental regulation on GTFP. Finally, this study uses provincial-level data, and its availability is limited. To be specific, due to the remarkable differences in economic development levels across different cities within a province, it is meaningful and reasonable to explore the relationship among environmental regulation, governance transformation, and the GTFP for different regions when city-level data are available in the future. Specifically, the use of city-level data can not only control the potential heterogeneity across cities within a province, but it can also enhance the credibility due to the significant expansion of the sample size.

## Figures and Tables

**Table 1:** Descriptive statistics of the variables from 2003 to 2017.

Variable	Definition	Obs	Mean	Min	Max	Std. Dev.
lnGTFP	Green total factor productivity	450	0.134	−0.115	0.690	0.168
lnER	Environmental regulation	450	2.259	−0.451	4.472	0.866
lnGT	Governance transformation	450	9.234	6.613	11.259	1.043
lnRD	R&D investment	450	4.00	1.104	5.054	0.494
lnEX	Export trade dependence	450	6.819	4.285	9.122	1.002
lnFDI	Foreign direct investment	450	5.112	1.351	6.958	1.301
ln(K/L)	Factor endowment structure	450	2.979	1.798	4.773	0.608

**Table 2:** Unit root and VIF test results.

Variable	LLC	IPS	Fisher-ADF	Fisher-PP	VIF
lnGTFP	−1.0309	−3.0823 **	97.7870 **	65.2958	—
lnER	−2.2471 *	−5.7744 ***	142.7996 ***	66.8284	1.60
lnGT	−7.3828 ***	−6.5908 ***	92.2760 **	94.7653 ***	2.22
lnRD	−5.4882 ***	−1.2160	181.8429 ***	114.186 ***	1.24
lnEX	−1.9065 *	−6.5586 ***	137.0833 ***	150.429 ***	1.79
lnFDI	−2.3105 **	−4.4187 ***	162.0403 ***	62.5083	1.84
ln(K/L)	−3.4148	−5.5994 ***	64.1777	128.726 ***	1.31

Note: ***, **, and * represent mean significance at the 1%, 5%, and 10% levels, respectively.

**Table 3:** The regression results of impact of environmental regulation on GTFP for full samples.

Variable	FGLS		FE		RE		DIFF-GMM		SYS-GMM	
	(1)	(2)	(3)	(4)	(5)	(6)	(7)	(8)	(9)	(10)
lnER	−0.0386 *** (0.0146)	−0.0377 *** (0.0141)	−0.0782 ** (0.0393)	−0.0708 * (0.0389)	−0.0765 * (0.0400)	−0.0685 * (0.0395)	−0.0556 *** (0.0217)	−0.0578 ** (0.0275)	−0.0749 ** (0.0354)	−0.0950 ** (0.0424)
(lnER)^2^	0.0038 (0.0037)	0.0045 (0.0037)	0.0076 (0.0091)	0.0068 (0.0089)	0.0063 (0.0092)	0.0066 (0.0089)	0.0097 * (0.0057)	0.0116 * (0.0070)	0.0100 * (0.0072)	0.0155 * (0.0094)
lnGT		0.0930 *** (0.0142)		0.0504 * (0.0316)		0.0512 ** (0.0222)		0.1277 *** (0.0432)		0.0269 * (0.0146)
lnRD	0.0691 *** (0.0132)	0.0726 *** (0.0144)	0.1025 *** (0.0296)	0.1114 *** (0.0270)	0.1077 *** (0.0256)	0.1051 *** (0.0239)	0.0831 *** (0.0306)	0.1232 *** (0.0389)	0.0924 *** (0.0290)	0.0742 *** (0.0265)
lnFDI	−0.0028 (0.0120)	−0.0043 (0.0108)	0.0117 (0.0203)	0.0084 (0.0202)	0.0142 (0.0183)	0.0082 (0.0192)	0.0232 (0.0177)	0.0153 (0.0191)	0.0188 (0.0149)	0.0120 (0.0133)
lnEX	−0.0019 (0.0144)	−0.0160 (0.0164)	0.0173 (0.0304)	0.0105 (0.0290)	0.0152 (0.0234)	0.0025 (0.0221)	0.0049 (0.0161)	0.0299 (0.0267)	−0.0224 * (0.0142)	−0.0367 * (0.0194)
ln(K/L)	0.0557 *** (0.0178)	−0.0046 (0.0179)	−0.0763 ** (0.0280)	0.0365 (0.0463)	−0.0661 ** (0.0262)	0.0358 (0.0326)	0.0422 * (0.0240)	−0.0278 (0.0425)	−0.0458 *** (0.0170)	−0.0550 *** (0.0166)
L.lnGTFP							0.3794 *** (0.1147)	0.2735 *** (0.1030)	0.6613 *** (0.0980)	0.6381 *** (0.1041)
Inflection point	5.0789	4.1889	5.1447	5.2058	6.0714	5.1894	2.866	2.491	3.745	3.065
R^2^	0.0955	0.0956	0.3799	0.3952	0.3790	0.3747				
AR (1)							0.0340	0.0469	0.0332	0.0299
AR (2)							0.4499	0.6814	0.4249	0.4253
Hansen							0.1062	0.1726	0.1811	0.1579
Observations	450	450	450	450	450	450	450	450	450	450

Note: standard errors in parentheses. ***, **, and * represent mean significance at the 1%, 5%, and 10% levels, respectively.

**Table 4:** Regression results of different regions with SYS-GMM.

Variable	Eastern Region		Central Region		Western Region	
	(1)	(2)	(3)	(4)	(5)	(6)
lnER	−0.0309 * (0.0168)	−0.0304 * (0.0199)	−0.0250 (0.0360)	−0.0410 (0.0419)	−0.1949 *** (0.0640)	−0.2207 *** (0.0571)
(lnER)^2^	0.0057 (0.0051)	0.0089 * (0.0051)	−0.0099 (0.0106)	0.0104 (0.0103)	0.0289 ** (0.0131)	0.0336 *** (0.0131)
lnGT		0.0202 ** (0.0092)		0.0621 *** (0.0176)		0.0215 * (0.0141)
lnRD	0.0772 ** (0.0352)	0.0380 ** (0.0170)	0.0303 ** (0.0137)	0.0285 (0.0286)	0.0829 ** (0.0364)	0.0849 * (0.0522)
lnFDI	0.0566 ** (0.0266)	0.0387 *** (0.0113)	0.0008 (0.0107)	−0.0074 (0.0150)	0.0059 (0.0100)	0.0104 (0.0117)
lnEX	−0.0501 * (0.0261)	−0.0510 *** (0.0187)	−0.0089 (0.0089)	0.0484 *** (0.0169)	0.0110 (0.0111)	-0.0074 (0.0148)
ln(K/L)	−0.0557 * (0.0335)	−0.0389 ** (0.0188)	−0.0117 (0.0187)	0.0104 (0.0137)	−0.0200 * (0.0110)	−0.0377 ** (0.0189)
L.lnGTFP	0.8112 *** (0.0837)	0.8520 *** (0.0609)	0.8335 *** (0.0913)	0.7040 *** (0.0807)	0.2630 ** (0.1369)	0.2151 ** (0.1164)
Inflection point	2.7105	1.7079	—	—	3.3720	3.2842
AR(1)	0.0133	0.0129	0.0430	0.0411	0.0820	0.0985
AR(2)	0.7593	0.7951	0.2102	0.2336	0.3001	0.3265
Hansen	0.1015	0.1258	0.2136	0.1300	0.5730	0.4749
Observations	165	165	120	120	165	165

Note: Standard errors in parentheses. ***, **, and * represent mean significance at the 1%, 5%, and 10% levels, respectively. The eastern region consists of Liaoning, Hebei, Tianjin, Beijing, Shandong, Jiangsu, Shanghai, Zhejiang, Fujian, Guangdong, and Hainan. The central region consists of Heilongjiang, Jilin, Shanxi, Anhui, Jiangxi, Henan, Hubei, and Hunan. The western region consists of Shanxi, Gansu, Ningxia, Qinghai, Xinjiang, Sichuan, Yunnan, Guangxi, Guizhou, Chongqing, and Inner Mongolia.

**Table 5:** Robustness test results of impact of environmental regulation on GTFP.

Variable	Panel Threshold Model		Replacing ER Variable		Two-step SYS-GMM		Adjusting Sample Interval	
	(1)Low Group	(2) High Group	(3)	(4)	(5)	(6)	(7)	(8)
lnER	−0.0564 ** (0.0274)	0.1727 * (0.1003)	−0.0768 ** (0.0354)	−0.1023 ** (0.0540)	−0.1811 ** (0.0874)	−0.1487 ** (0.0715)	−0.0818 ** (0.0356)	−0.1404 ** (0.0666)
(lnER)^2^			0.0096 * (0.0066)	0.0159 * (0.0085)	0.0322 * (0.0181)	0.0269 ** (0.0141)	0.0133 * (0.0080)	0.0262 * (0.0147)
lnGT	0.1450 *** (0.0473)	0.1107 ** (0.0547)		0.0265* (0.0149)		0.0513 ** (0.0247)		0.0103 (0.0144)
lnRD	0.1682 *** (0.0462)	0.1093 ** (0.0504)	0.0923 *** (0.0297)	0.0751 ** (0.0277)	0.1030 ** (0.0436)	0.0864 * (0.0470)	0.0866 ** (0.0389)	0.0901 ** (0.0470)
lnFDI	0.0209 (0.0326)	0.0107 (0.0367)	0.0206 (0.0151)	0.0144 (0.0135)	0.0092 (0.0171)	0.0019 (0.0146)	0.0388 (0.0367)	0.0346 (0.0281)
lnEX	−0.1595 *** (0.0579)	−0.1899 *** (0.0567)	−0.0224 (0.0149)	−0.0367 * (0.0197)	−0.0116 (0.0111)	−0.0351 (0.0275)	−0.0414 (0.0275)	−0.0429 ** (0.0184)
ln(K/L)	−0.1937 *** (0.0589)	−0.2215 ** (0.0891)	−0.0443 ** (0.0179)	−0.0525 *** (0.0166)	−0.0313 (0.0295)	−0.0339 * (0.0207)	−0.0350 ** (0.0176)	−0.0418 *** (0.0142)
L.lnGTFP	0.3303 ** (0.1586)	0.6457 *** (0.1174)	0.5854 *** (0.1405)	0.6369 *** (0.1046)	0.5716 *** (0.1152)	0.5569 *** (0.1286)	0.7413 *** (0.1275)	0.7233 *** (0.1485)
Inflection point	3.150 (threshold value)		4.000	3.217	2.812	2.764	3.0752	2.6794
AR (1)	0.0078		0.0317	0.0280	0.0259	0.0194	0.0588	0.0454
AR (2)	0.1964		0.4294	0.4192	0.4346	0.3971	0.3303	0.3166
Hansen	0.6852		0.1922	0.3304	0.2441	0.2910	0.1224	0.4002
Observations	450		450	450	450	450	390	390

Note: Standard errors in parentheses. ***, **, and * represents mean significance at the 1%, 5%, and 10% levels, respectively.

**Table 6:** The regression results of impact of governance transformation on the Porter hypothesis.

Variable	SYS-GMM						DIFF-GMM	
	(1)	(2)	(3)	(4)	(5)	(6)	(7)	(8)
lnER	−0.2481 *** (0.0654)	−0.2732 *** (0.0730)	−0.3034 *** (0.0872)	−0.2756 *** (0.0777)	−0.2258 *** (0.0670)	−0.2498 *** (0.0649)	−0.2408 (0.1884)	−0.5657 *** (0.2176)
(lnER)^2^	0.0155 * (0.0092)	0.0148 * (0.0085)	0.0153 * (0.0085)	0.0151 ** (0.0077)	0.0135 ** (0.0072)	0.0142 * (0.0080)	0.0189 * (0.0101)	0.0325 ** (0.0148)
lnGT	0.0178 *** (0.0052)	0.0078 (0.0053)	0.0273 ** (0.0137)	0.0329 ** (0.0127)	0.0089 (0.0180)		0.0982 ** (0.0500)	
lnER*lnGT	0.0182 *** (0.0060)	0.0212 *** (0.0063)	0.0236 *** (0.0074)	0.0226 *** (0.0065)	0.0165 *** (0.0057)	0.0187 *** (0.0048)	0.0295* (0.0165)	0.0444** (0.0180)
lnRD		0.0615 *** (0.0191)	0.0785 *** (0.0244)	0.0809 *** (0.0218)	0.0828 *** (0.0250)	0.0879 *** (0.0285)	0.1319 *** (0.0441)	0.1316 *** (0.0494)
lnFDI			0.0250 * (0.0138)	0.0203 (0.0158)	0.0016 (0.0143)	0.0027 (0.0140)	0.0149 (0.0175)	0.0179 (0.0179)
lnEX				0.0102 (0.0156)	−0.0183 (0.0229)	−0.0109 (0.0158)	0.0385 (0.0255)	0.0364 (0.0240)
ln(K/L)					−0.0428 ** (0.0209)	−0.0392 ** (0.0176)	−0.0178 (0.0464)	−0.0075 (0.0485)
L.lnGTFP	0.6306 *** (0.0928)	0.5937 *** (0.0999)	0.5738 *** (0.0948)	0.5801 *** (0.0867)	0.5852 *** (0.0910)	0.5831 *** (0.0894)	0.1862 * (0.1106)	0.2348 * (0.1233)
AR (1)	0.0434	0.0363	0.0296	0.0354	0.0389	0.0373	0.0221	0.0129
AR (2)	0.4082	0.4200	0.4027	0.4238	0.4369	0.4363	0.8091	0.5174
Hansen	0.3387	0.5999	0.5471	0.5183	0.3637	0.4029	0.1211	0.1461
Observations	450	450	450	450	450	450	450	450

Note: Standard errors in parentheses. ***, **, and * represent mean significance at the 1%, 5%, and 10% levels, respectively.

## Data Availability

The datasets and computer programs used in this study are available from the corresponding author upon reasonable requests.
